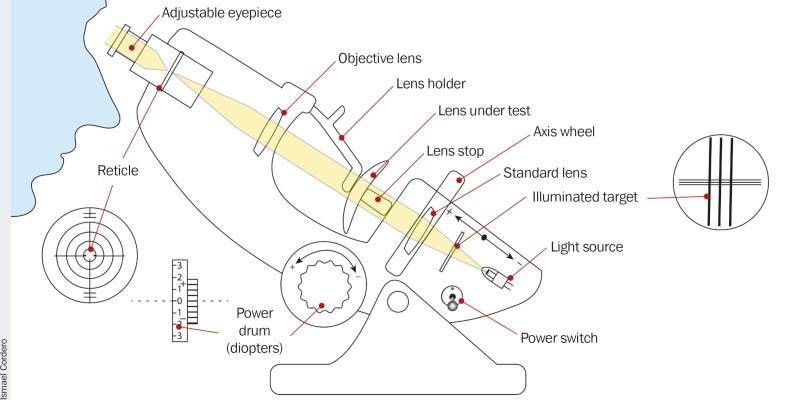# Understanding and caring for a lensmeter

**Published:** 2016

**Authors:** Ismael Cordero

**Affiliations:** Clinical Engineer, Philadelphia, USA. **ismaelcordero@me.com**

**Figure:**
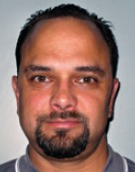
Ismael Cordero

A lensmeter or lensometer is an instrument used to verify the prescription of eyeglasses or spectacles. Many lensmeters can also verify the power of contact lenses with the addition of a special lens support. The values obtained from a lensmeter are the values specified on the patient's eyeglass prescription: sphere, cylinder, axis, add, and in some cases, prism. It is commonly used prior to an eye examination to obtain the last prescription the patient was given, in order to expedite the examination.

In one commonly used type of lensmeter the target seen through the eyepiece consists of a set of three wide lines with wide spacing between them and another set of three narrow lines with smaller spacing between them. These two sets of lines intersect at right angles. The closely spaced lines represent the sphere component of lens power and the thicker, widely spaced lines represent the cylinder power. In the case of a spherical lens, all of the lines of the target will focus at the same time, while in the case of a sphero-cylindrical lens, the lines will focus separately at different power drum readings (see illustration).

In another type of lensmeter, a series of light dots forming a circle is used as a target instead of the two sets of parallel lines described earlier. If a spherical lens is measured, the circle remains a circle and the power drum is adjusted to obtain a sharp image of the dots. For sphero-cylindrical lenses the dots, when focused, will display a sharp ellipse. The major and minor axes of the ellipse can be read on the scale provided in the instrument.

The target is imaged through a lens. The eyeglass lens under test is placed at the rear focal point of this lens. Light emerging from the spectacle lens enters the eyepiece which contains a reticle. The reticle is a permanently etched series of concentric rings used to measure and locate the prism base direction, and also contains orientation lines for each lens meridian and a protractor scale.

For measuring the power of the lens, the power drum is turned until a clear and sharp image of the target is seen through the eyepiece. The power (in diopters) can be read on the scale on the wheel. For measuring the focal power of cylindrical and sphero-cylindrical lenses that have different powers in different meridians, the optics of the equipment can be rotated by turning the axis wheel. The angular position can be read on the circular scale of the axis wheel.

The eyeglass lens to be tested should be placed on the lens stop so that the outside of the lens is facing the eyepiece and the side of the lens that sits closest to the user's eye is facing the instrument's light source.

Before using the instrument, you should look through the eyepiece. The reticle should be in focus. If it is not, adjust the eyepiece until it is sharply focused.

## Checking power calibration

Periodically ensure that the power calibration of your lensmeter is accurate by following these steps:

- Turn on the lensmeter.
- Turn the eyepiece ring so that the reticule appears in focus.
- Turn the power wheel into the plus, then slowly decrease the power until the lensmeter target is sharply focused. Do not oscillate the wheel back and forth to find the best focus. The power wheel should read zero if the instrument is properly calibrated.
- If the power wheel does not read zero, re-focus the eyepiece and re-check the calibration. If the power wheel still does not read zero, the error must be compensated for in all future measurements made with the lensmeter, or the lensmeter needs maintenance. (Note: subtract the calibration error from the power measurement to compensate for calibration errors.)

## General care

- If dust falls onto the surface of the lenses of the lensmeter, blow it off with a clean bulb syringe, a dust brush or compressed air.
- Wipe the instrument's exterior with a soft cloth to prevent dust accumulation.
- Do not attempt to lubricate the instrument. If it feels tight, contact a qualified service technician.
- To prolong the life of the bulb, do not leave the instrument on all day.
- Cover the lensmeter when not in use to protect it from dust.

**Figure 1:**